# Design and Fabrication of Artificial Stem Cell Microenvironments

**DOI:** 10.3390/bioengineering9120756

**Published:** 2022-12-02

**Authors:** Panagiotis Mallis

**Affiliations:** 1 Hellenic Cord Blood Bank, Biomedical Research Foundation Academy of Athens, 4 Soranou Ephessiou Street, 115 27 Athens, Greece; pmallis@bioacademy.gr or p.mallis@gna-gennimatas.gr; 2 Immunology Department & National Tissue Typing Center, General Hospital of Athens “Gennimatas”, 154 Mesogeion Ave., 115 27 Athens, Greece

Major key features of stem cells’ functions are self-renewal and their capacity for differentiation, allowing for maintain a proper stem cell reservoir as well as producing lineage-committed cells [1].

These unique abilities of stem cells are mediated through asymmetric cell division, which eventually leads to the production of a stem cell and a progenitor cell that will further give rise to downstream cellular populations [1,2,3]. The mechanism by which the stem cells can differentiate plays an essential role in organogenesis during the different stages of embryonic development [4,5]. Moreover, stem cell fate is orchestrated by the presence of bivalent epigenetic modifications [6,7]. Indeed, the presence of both H3K4me3 and H3K27me3 is implicated in the transcriptional control of gene repression or activation [6,7]. On the other hand, stem cells in adults play a pivotal role in tissue homeostasis, contributing either to the generation of progenitor cells in order to accelerate the recovery of damaged tissue or to produce specific cell types that will serve different system functions, e.g., T and B cells in adaptive immunity, red blood cells in peripheral blood, etc. [8,9].

The determination of cell fate is regulated by extrinsic signals that will eventually promote specific intrinsic cell programs [10]. These extracellular stimuli are mostly originated from the niche, based on specific genetic directions and organism’s requirements [11]. To maintain the proper function of the organism, a controlled balance between the self-renewal and differentiation of stem cells must exist. Indeed, uncontrolled stem cell proliferation or impaired cell differentiation may be related to major human diseases such as hematological malignancies and autoimmune disorders [12]. Moreover, the study of the related mechanism deciding a stem cell’s fate may shed light on the “cause and causality” aspects of disease, such as the underlying intrinsic mechanisms of cancer development, tumor growth and metastasis. Beyond these events, there is increasing evidence that specific stem cells can escape from their niche in order to contribute to tissue’s homeostasis. A great example of the latter is the Mesenchymal Stromal Cells (MSCs), a multipotent stem cell population which, in adults, can be found in several tissues such as bone marrow (BM), adipose tissue, the liver, lungs, etc. [13]. MSCs are well known as the BM stroma that regulate the differentiation of Hematopoietic Stem Cells (HSCs). However, under specific conditions such as in immune responses towards a pathogen, BM-MSCs can migrate to inflamed tissue through chemokine gradients produced by the professional antigen-presenting cells (APCs) [13,14]. Once arriving at the site of inflammation, MSCs can be primed through the paracrine action of interferon-γ (IFN-γ), produced by activated macrophages and T cells, and elevate the expression of human leukocyte antigen (HLA) class II and co-stimulatory molecules in order to take part in antigen presentation [13,14]. In this way, the primed MSCs can further promote immune responses, through specific communication with the cells of both innate and adaptive immunity [13,14].

In the context of controlling stem cell behavior, the niche plays a crucial role in either maintaining the cells in a “poised” (G0) state or controlling their proper proliferation and differentiation [1,2,3,4]. Moreover, improving our understanding of how the niche can regulate stem cell fate may contribute to better cell administration in different disorders as a potential “advanced stem cell therapy” [15]. Under this scope, the design, development and fabrication of artificial stem cell microenvironments are highly demanding tasks which need to be explored. For this purpose, many research groups are currently focused on designing and producing topographically controlled biomaterials that can potentially mimic the stem cell niche [16]. In such a way, lithography, electrospinning and 3D bioprinting represent state-of-the-art methods for the production of biomaterials sufficient to mimic the stem cell niche [17]. Moreover, the next-generation bioprinting method, so-called “4D Bioprinting”, enables the development of smart materials with shape-shifting properties and has attracted the attention of the scientific community for the manufacturing of artificial microenvironments. Fabricated 4D-printed smart materials, upon exposure to external stimuli such as pH, heat, magnetic field, light and humidity, can effectively change their shape, accompanied by different material properties [17].

This Special Issue aimed to gather novel approaches in the design and production of functional and synthetic microenvironments in order to better study the specific association patterns between stem cells and their niche.

The stem cell niche consists of key components such as specific extracellular matrix (ECM) proteins (e.g., collagen type I and II; proteoglycans including versican, perlecan and aggrecan; and sulfated glycosaminoglycans such as chondroitin sulfate, chondroitin and heparin sulfate) that can drive specific stimuli cues to the resident stem cells [16]. The term “niche” was introduced in 1978 by Schofield in an effort to explain the self-renewal ability of HSCs [18]. Stem cells within their niche are subjected to various direct or indirect interactions exerted by both neighboring cells (stem cell niche stroma) and ECM-mediated signals, such as those obtained from shear stress and tissue stiffness [16,18,19].

Many niches beyond the first described HSC niche have been investigated to date, such as the neural stem cells (NSCs) niche and the olfactory bulb and hair follicle niches [16,19]. In addition to the existing differences between these niches, some common features have been described in the literature [16,19]. These mostly include the presence of (a) heterologous cell–cell interaction, (b) secreted growth factors, (c) immunological regulation during inflammation or tissue damage and (d) ECM-mediated interactions with the stem cells. In this way, specific signaling pathways can effectively modulate the self-renewal or proliferation of stem cells. For example, the Wnt signaling pathway effectively regulates the fate of HSCs through its related downstream pathway (Wnt–Wnt receptor, activation of GSK3β/Axin and nuclear β-catenin), which can further modulate their self-renewal state or differentiation potential [20].

Accordingly, and based on the above stem cell niche features, designing and fabricating artificial niches represents a highly demanding task. It is of major importance to properly design the artificial stem cell niche in order to efficiently control the stem cell fate under in vitro culture conditions. In this way, artificially developed niches can be used to study different aspects of stem cell features in order the precision of “advanced stem cell therapy” to be further promoted in clinical utility (Figure 1).

The investigation of “niche-mediated” interactions with the different types of stem cells may shed light on significant aspects of life and disease. However, the structure–function properties of the niche are closely related to the behavior exerted by the stem cells; hence, further exploration of their properties is required.

In the context of artificial stem cell niche development, Ramos-Rodriguez et al. [21,22] presented different microfabrication techniques for their proper production. As has been previously mentioned, the stem cell niche is an emerging field of research, which has received great attention by the research society in the last century. The functions of both embryonic and adult stem cells are mostly driven by specific stimuli cues received by their niche. Hence, mimicking the structure and developing a complete “off-the-shelf” artificial microenvironment represents a great challenge. To address these issues, research groups are focused on the use of different manufacturing techniques to mimic the physical features of the stem cell niche and to properly investigate the cell responses towards different stimuli [21,22]. To achieve this goal, manufacturing methods such as soft-lithographic approaches, electrospinning and 3D printing have been proposed for artificial niche development. Specifically, soft-lithographic approaches utilize elastomeric stamps, different molds and photomasks (from the micrometer to nanometer scale) in order to create different patterned surfaces. Lithography methods, including photolithography and stereolithography, can create high-resolution structures (100 nm–10 μm). Moreover, 3D printing methods currently represent the state-of-the-art approaches, utilizing synthetic or natural materials to build highly detailed structures. Moreover, the fabricated structures can be further modified to include Arg-Gy-Asp (RGD) peptides, immobilized growth factors and proteoglycans in order to achieve better cell attachment, proliferation and differentiation [21,22]. To, Microfluidics devices have been broadly used to better study 3D-fabricated organized structures. Microfluidics systems can control specific fluids on a micrometer to millimeter scale, which further allows the precise control of soluble factors and mechanical signals in both natural and artificial stem cell microenvironments. Recently, microfluidics devices have been applied in the development of high-throughput cell culture systems and organs-on-a-chip, thus allowing the comprehensive study of single-cell interactions [21,22].

Further study regarding “stem cell niche-mediated” interactions have been performed by a number of researchers that provided substantial contributions to this Special Issue organized by Dr Ilida Ortega Asencio and Dr Farshid Sefat. More specifically, Liu et al. [23], in their study, presented the successful development of synthetic tracheal grafts modulated with ECM components, to better support better cell adhesion to the matrix surface. The trachea scaffolds were developed using a composition of different materials such as polyethene terephthalate (PET) and polyurethane (PU) in 1,1,1,3,3,3-hexafluoroisopropanol (HFIP). The obtained solution was electrospun on a flat sheet of 2 mm x 5 mm, finally leading to the development of cylindrical grafts [23]. Then, the produced trachea scaffolds were coated with ECM substrates, including fibrillin, laminin, collagen and nephronectin, derived from decellularized-lyophilized human lungs. The results of this study clearly showed better adhesion and proliferation of the lung epithelial cell line (A549) in coated trachea scaffolds compared to uncoated scaffolds. Moreover, it was proven that lung-derived ECM components were nontoxic and promoted cell attachment on the trachea scaffolds in a dose-dependent manner [23].

One of the most important and first described microenvironments was the Hematopoietic Stem Cell (HSC) niche, which was comprehensively reviewed by Geoffrey Brown in this Special Issue [24]. HSCs represent heterogeneous populations of cells, and their niche, also referred to as the “endosteal niche”, is located in the internal bone shell surface, close to the endocortical and trabecular surfaces. This endosteal region of the bone marrow is highly perfused by arterioles and arterial capillaries, where quiescent HSCs reside around these structures. Specific signaling cues originated by the perivascular cells, endothelial cells, Schwann and sympathetic neuronal cells can control the HSCs fate. In addition to the aforementioned cells, the HSC niche also includes osteoblasts, chondroblasts, adipoblasts and MSCs, which also play crucial roles in HSC fate and in an organism’s homeostasis. The differentiation of HSCs gives rise to hematopoietic progenitor cells (HPCs), which in turn lead to the production of all mature myeloid- and lymphoid-derived blood cells [24]. Moreover, the determination of HSC fate is accomplished by a combination of intrinsic and extrinsic signaling cues originated by both stroma interactions and a significant set of growth factors. The HSCs’ quiescence and self-renewal are controlled by the interaction of the previously mentioned cells with the stroma cells through CXCL12 and CXR4. In addition, growth factors such as M-CSF, G-CSF, GM-CSF, EPO and Flt3L can regulate the balance between self-renewal and differentiation/proliferation [24]. In addition to the above functions, the HSC niche holds a crucial role in clearing out leukemia stem cells (LSCs). Recent evidence supports that LSCs are lineage-restricted to HSCs, which are characterized by aberrant oncogene or tumor suppressor gene function. However, more experimental research is required to better study best practices for decision makers in the determination of HSCs’ fate in health and disease [24].

To better understand the functional association between niche and stem cells, Alvarez et al. [25] and Abdul-AL et al. [26] explained the significance of artificial stem cell niches in the anterior ocular segment. Currently, regenerative medicine has provided significant solutions for the control of multiple pathological events on the ocular surface, which can lead to long-term vision loss. For this purpose, ocular stem cell therapy and ex vivo production of cornea and conjunctival tissues have already been used with great success. However, a significant remaining challenge is the preservation of stem cell features under in vitro culture conditions. For this reason, an artificial microenvironment mimicking the natural stem cell niche that may favor the physicochemical properties and dynamic cell–extracellular matrix interactions is needed, which will further support the stemness properties [25,26]. In this way, the biofabrication of stem cell niches for the anterior ocular segment can be achieved utilizing advanced manufacturing methods such as photolithography, electrospinning and 3D bioprinting, as has been described previously [21,22]. Moreover, investigating the properties of the artificially produced ocular niches seeded with corneal-derived cells, such as corneal epithelial cells (CECs), limbal epithelial stem cells (LESCs) and corneal stromal stem cells (CSSCs), has been proposed and performed. By successfully combining the above stem cell populations with artificially produced ocular niches, new horizons regarding their use in regenerative medicine applications may be explored.

The data obtained from the investigation of natural stem cell niches can be potentially applied for the proper design and manufacturing of artificial microenvironments. In this way, and under in vitro culture conditions, the specific “niche–stem cell” associations can be better evaluated at a single-cell level. Alongside the state-of-the-art biofabrication approaches, artificial stem cell niches will play a crucial role in the future in helping us to better understand the stem cells’ functions and behavior. This may lead to new horizons in efficiently manipulating both stem cells and suitable artificial microenvironments and enabling the collection of further evidence regarding the onset of various diseases. Equipped with such evidence, modern therapeutic approaches can be proposed, bringing precision medicine one step closer to its clinical application.

## Figures and Tables

**Figure 1:**
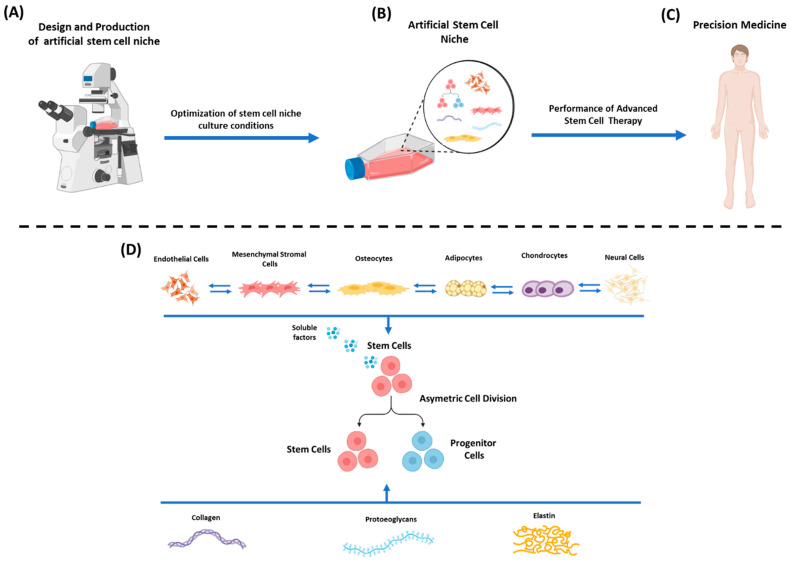
Design and fabrication of an artificial stem cell niche. (**A**) Design and production of artificial stem cell niche using state-of-the-art approaches (lithography, electrospinning, 3D printing). (**B**) Optimization of artificial stem cell niche in in vitro culture conditions. (**C**) Administration of stem cells as a potential “advanced cellular therapy” to the patient, promoting precision clinical medicine. (**D**) Functional artificial stem cell niche relies on the stimuli cues (through direct contact or soluble factors secretion) exerted by neighboring cells (e.g., endothelial cells, MSCs, osteocytes, adipocytes, chondrocytes, neural cells, etc.) and from ECM components (collagen fibers, proteoglycans, elastin, etc.). Specific cellular communication exists between all the cells of the niche in order to properly regulate their functions. The above elements can control stem cell fate by either promoting self-renewal or differentiation.
